# Electroacupuncture at Zusanli Prevents Severe Scalds-Induced Gut Ischemia and Paralysis by Activating the Cholinergic Pathway

**DOI:** 10.1155/2015/787393

**Published:** 2015-09-10

**Authors:** Huan Wang, Lei Wang, Xian Shi, Song Qi, Sen Hu, Zhangqi Tong, Zhuhong Ma, Yan Qian, Daniela Litscher, Gerhard Litscher

**Affiliations:** ^1^Department of TCM and Acupuncture, Chinese PLA General Hospital, Beijing 100853, China; ^2^Shock and Multiple Organ Dysfunction, Burns Institute, First Hospital Affiliated to The PLA General Hospital, Beijing 100048, China; ^3^Department of Traditional Chinese Medicine, Chinese PLA General Hospital, Beijing 100853, China; ^4^Department of Oncology, Chinese PLA General Hospital, Beijing 100853, China; ^5^Research Unit for Complementary and Integrative Laser Medicine, Research Unit of Biomedical Engineering in Anesthesia and Intensive Care Medicine, and TCM Research Center Graz, Medical University of Graz, 8036 Graz, Austria

## Abstract

Severe burn injuries may result in gastrointestinal paralysis, and barrier dysfunction due to gut ischemia and lowered vagus excitability. In this study we investigate whether electroacupuncture (EA) at Zusanli (ST36) could prevent severe scalds-induced gut ischemia, paralysis, and barrier dysfunction and whether the protective role of EA at ST36 is related to the vagus nerve. 35% burn area rats were divided into six groups: (a) EAN: EA nonchannel acupoints followed by scald injury; (b) EA: EA at ST36 after scald injury; (c) VGX/EA: vagotomy (VGX) before EA at ST36 and scald injury; (d) VGX/EAN: VGX before EAN and scald injury; (e) atropine/EA: applying atropine before scald injury and then EA at ST36; (f) atropine/EAN: applying atropine before scald injury and then EA at nonchannel acupoints. EA at the Zusanli point significantly promoted the intestinal impelling ratio and increased the amount of mucosal blood flow after scald injury. The plasma diamine oxidase (DAO) and intestinal permeability decreased significantly after scald injury in the EA group compared with others. However, EA after atropine injection or cervical vagotomy failed to improve intestinal motility and mucosa blood flow suggesting that the mechanism of EA may be related to the activation of the cholinergic nerve pathway.

## 1. Introduction

Patients with severe trauma and burns often suffer from gastrointestinal ischemia and paralysis, which may result in intestinal barrier dysfunction and remote multiple organ failure. However, at present, there is still a lack of effective drugs or interventions to prevent gut ischemia and paralysis and to protect the gut epithelial barrier which is very important to medical support for ischemia patients. It is known that neuronal networks representing physiological mechanisms can be exploited for the treatment of inflammatory and infectious disorders [1]. Recent studies showed that electrical stimulation of the vagus nerve could protect the intestinal barrier and alleviate inflammatory injuries in the intestines and remote organs of animals following burn injuries by activation of the cholinergic anti-inflammatory pathway [2, 3]. However, it is still difficult to apply electrical stimulation to the vagus nerve in clinical practice due to complicated manipulation and untoward side effects, including serious tissue injury. Traditional Chinese medicine, especially acupuncture, has made many important contributions to medicine. In China and neighboring countries/regions, it has served as the basis of medical knowledge for thousands of years. The acupuncture of Zusanli (ST36) points has been widely used in clinical practice to improve gastrointestinal dysfunction and cure some kinds of gastrointestinal diseases. Studies also showed that acupuncture at Zusanli points has the effect of regulating gastrointestinal function by promoting gastrointestinal motility and attenuating local inflammatory response [4–6]. However, much less is known regarding acupuncture effects on gastrointestinal dysfunction after burn injury, and more research is needed. It was reported that electroacupuncture (EA) could control systemic inflammation by inducing vagal activation of aromatical-amino acid decarboxylase, leading to the production of dopamine in the adrenal medulla. Since the effect of acupuncture at ST36 is similar to that of activating the vagus nerve [2, 3], we hypothesize that acupuncture at bilateral ST36 might exert a positive effect on gastrointestinal motility and mucosal blood flow, through stimulation of the vagus nerve pathway. Therefore, in the present study, we investigated the effect of EA at ST36 on the intestinal epithelial barrier and motility in scald injury rats and examined whether this effect might be based on the activation of the vagus nerve.

## 2. Materials and Methods

### 2.1. Rat Scald Model

120 male Wistar rats (7-8 weeks old, 230–250 g) were purchased from the Experimental Animal Center of Military Medical Sciences of the Chinese People's Liberation Army. The rats were housed in mesh cages in a room maintained at 25°C, illuminated with 12 : 12 hour light-dark cycles, and provided with standard rodent chow and water ad libitum. Scald injury comprising 35% of the total body surface area (TBSA) was produced as described by Ikezu et al. [7]. Briefly, rats were treated by immersing the back of the trunk for 15 seconds in 96°C water under anesthesia with pentobarbital sodium (50 mg/kg, Sigma-Aldrich, St. Louis, MO, USA). Sham-scalded rats were immersed into water at room temperature. After scald injury, animals received a subcutaneous injection of 0.2 mL normal saline (NS) with 0.1 mg/kg buprenorphine (Sigma-Aldrich) for pain control. After the experiments the animals were sacrificed. All animal experiments were approved by the Committee of Scientific Research of the First Hospital Affiliated to General Hospital of People's Liberation Army, Beijing, China, and were conducted in accordance with the National Institute of Health Guide for the Care and Use of Laboratory Animals.

### 2.2. Animal Grouping and Treatments

Experimental rats were randomly assigned to six groups (*n* = 20 each) after scald injury.

#### 2.2.1. EAN Group

Rats received EA at nonchannel acupoints (which is approximately 3 cm distal from the ST36 acupoint toward the tail and opposite to the knee joint. It is located over the semitendinosus muscle at 5 mm from the tail base. This nonacupoint is neither referred in the acupoint map of rodents nor referred close to any major nerve [3]) immediately after injury; EA parameters were the same as in the EA group.

#### 2.2.2. EA Group

Animals underwent EA at ST36 points, which are located at the posterior and lateral side of the knee joint, 5 mm below capitulum fibulae [8], immediately after the scald injury. EA at ST36 was performed using an electroacupuncture apparatus (HANS, made in China, LH202H) as described before [6], with an intensity of 2 mA and a frequency of 2–100 Hz, for approximately 1.5 hours immediately after scalds.

#### 2.2.3. VGX/EA Group

Animals underwent vagotomy of the dorsal and ventral vagus nerve on the distal esophagus prior to EA at ST36 points immediately after scald injury; EA parameters were the same as in the EA group.

#### 2.2.4. VGX/EAN Group 

Animals underwent vagotomy similar to the VGX/EA group before EA at nonchannel acupoints (similar to EAN group) immediately after scald injury. EA parameters were the same as in the EA group.

#### 2.2.5. Atropine/EA Group

An intraperitoneal injection of atropine (2 mg/kg) was administered prior to the scald injury and followed by EA at ST36 (similar to EA group). EA parameters were the same as in the EA group.

#### 2.2.6. Atropine/EAN Group

An atropine intraperitoneal injection was administered prior to scald followed by EA at nonchannel acupoints (similar to EAN group).

Each group was divided into four subgroups (each *n* = 5). The rats were sacrificed at −0.5 h, 0 h, 2 h, and 6 h after scald, respectively, to detect the mucosal blood flow in the small intestine, intestinal motility, intestinal epithelial permeability, and diamine oxidase (DAO).

### 2.3. Intestinal Propulsive Rate and Mucosal Blood Flow

2% blue dextran 2000 (BD-2000) solution (GE Healthcare company, Sweden) was injected into the small intestine by an 85 mm gavage needle at 10 mL/kg. 30 min later, the animals were anesthetized with inhaled isoflurane and given a 3 cm abdominal incision through the ventral midline. Then we detected the mucosal blood flow in the small intestine by placing the laser Doppler probes (PeriFlux5000, PERIMED company, Sweden) directly on the pylori. At last we drew blood samples from the abdominal aorta before sacrificing the animals. We measured the length (*L*1) of the intestine (from pylorus to epityphlon) and the advance distance (*L*) of BD-2000, using the pylorus as base point, by separating and stretching the intestine on white paper. The BD-2000 intestinal impelling ratio (%) was calculated as = *L*/*L*1 × 100%.

### 2.4. DAO Activity Test

The freshly extracted blood was centrifuged for 10 min at 3000 rpm. 80 *µ*L supernatant were mixed with 800 *µ*L Tris-HCl (PH 7.4, NADH, GLDH). Then the mixture was poured into a Quartz cuvette and the result OD1 was obtained from a UV spectrophotometer working with a wavelength of 340 nm after 20 seconds. The mixture was then poured into a water bath for 10 min and the result OD2 was obtained from the UV spectrophotometer after 620 seconds. Taking OD1 and OD2 into the formula, the activity of DAO was calculated as {(OD1 − OD2)/(10 × 0.5 × 6.3)}×(880/80) × 1000, and the enzyme activity was measured.

### 2.5. Intestinal Epithelial Permeability

An in vivo intestinal permeability assay was performed to assess gut barrier function as described by Kao et al. [9]. Briefly, 30 min before sacrifice, animals were anesthetized with inhaled isoflurane. A midline laparotomy incision was performed and a 10-cm segment of the distal ileum was isolated between silk ties. A solution of 1.0 mL phosphate-buffered saline (PBS, pH 7.2) containing 25 mg 4-kDa fluorescein isothiocyanate- (FITC-) dextran (Sigma-Aldrich, St. Louis, MO, USA) was injected into the lumen of the isolated segment of the intestine. The bowel was returned to the abdominal cavity and the abdomen was closed. Animals were maintained under light general anesthesia for 30 minutes, at which time systemic blood was drawn by left femoral artery puncture and placed in heparinized Eppendorf tubes on ice. Plasma was obtained by centrifuging the blood at 10,000 g for 10 minutes at −4°C. Plasma fluorescence was measured in a fluorescence spectrophotometer (Synergy2; BioTek Multi-Detection Microplate reader, USA) and compared with a standard curve of known concentrations of FITC-dextran diluted in rat plasma.

### 2.6. Statistical Analysis

SPSS 13.0 statistical software was used, and all results were expressed as mean ± SD. One-way analysis of variance was used for comparison among all the groups, followed by the Student-Newman-Keuls test for comparison between two groups. Differences were considered to be statistically significant when *P* < 0.05.

## 3. Results

### 3.1. Effect of EA at ST36 on the Intestinal Impelling Ratio

**Figure 1:**
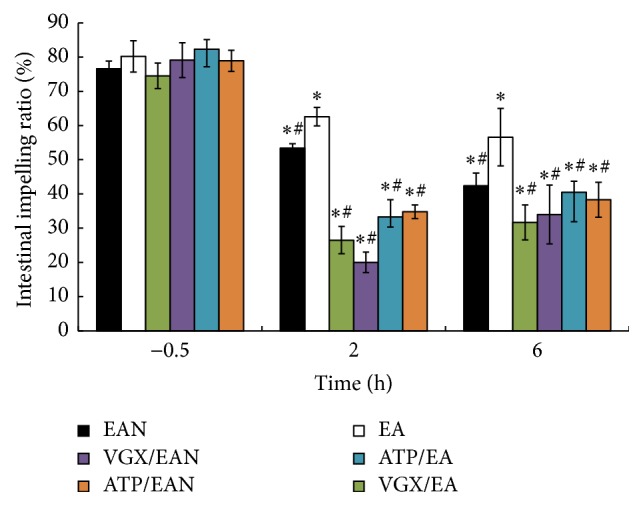
Intestinal impelling ratio was detected at −0.5, 2, and 6 h after scalds injury. Data are expressed as means ± SD (*n* = five animals at every time point per group). *∗* versus −0.5 h among the same group, *P* < 0.05; # versus EA group among 2 h and 6 h, *P* < 0.05.

Figure 1 illustrates the effect of EA at ST36 on the intestinal impelling ratio after 35% of TBSA scald injury. Scald injury induced gut paralysis and decreased the intestinal impelling ratio. EA at ST36 increased the intestinal impelling ratio after scald injury, while EA at nonchannel acupoints, vagotomy, or intraperitoneal injection of atropine before EA at ST36 reversed its antiparalysis effects. This evidence suggests that EA at ST36 attenuates the decrease of the intestinal impelling ratio after scalds injury.

### 3.2. Effect of EA at ST36 on the Mucosal Blood Flow in the Small Intestine

**Figure 2:**
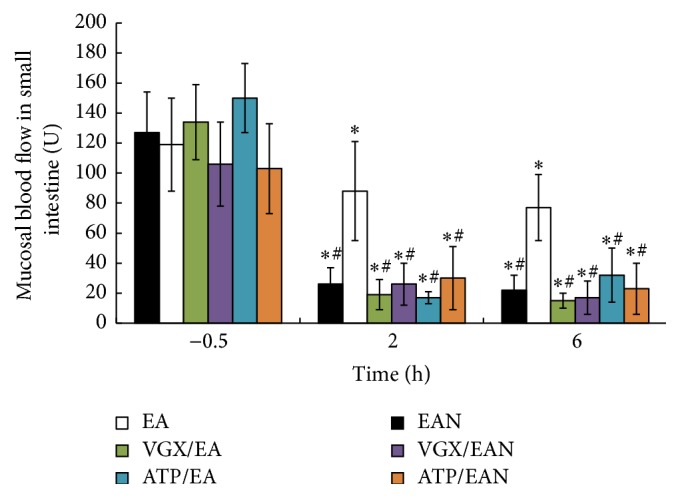
Mucosal blood flow in the small intestine was detected at −0.5, 2, and 6 h after scalds injury. Data are expressed as means ± SD (*n* = five animals at every time point per group). *∗* versus −0.5 h among the same group, *P* < 0.05; # versus EA group among 2 h and 6 h, *P* < 0.05.

Figure 2 illustrates the effect of EA at ST36 on the mucosal blood flow in the small intestine after 35% of TBSA scald injury. Scald injury induced gut ischemia. EA at ST36 increased the small intestinal mucosal blood flow after scalds injury, while EA at nonchannel acupoints, vagotomy, or intraperitoneal injection of atropine before EA at ST36 reversed its anti-ischemic effects. This evidence suggests that EA at ST36 attenuates the decrease of mucosal blood flow in the small intestine after scalds injury.

### 3.3. EA at ST36 Lowered the Intestinal Permeability

**Figure 3:**
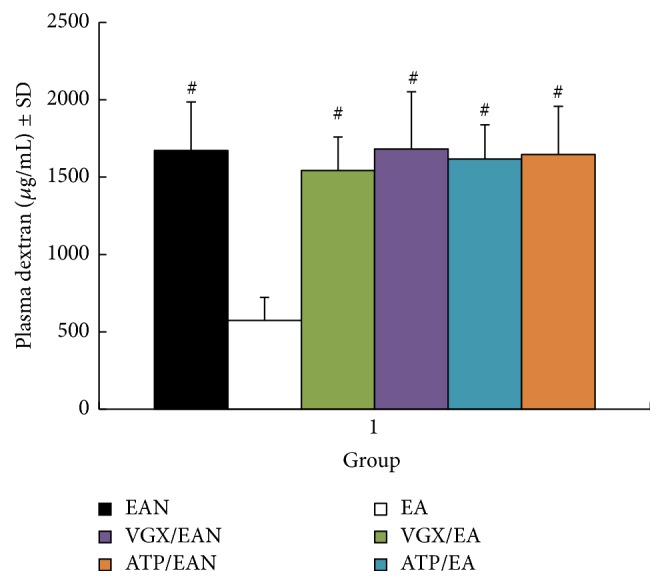
The intestinal permeability was detected in an in vivo assay 6 h after the scalds injury. Data are expressed as means ± SD (*n* = five animals at every time point per group). # versus EA group, *P* < 0.05.

The intestinal permeability was evaluated in an in vivo assay using FITC-dextran 6 h after the scald (Figure 3). Animals in the EA group had a significantly lower level of plasma FITC-dextran when compared with the EAN group (574.31 ng/mL ± 149.45 ng/mL versus 1671.26 ng/mL ± 315.13 ng/mL, *P* < 0.05). However, when abdominal vagotomy or atropine injection was performed before EA at ST36, the intestinal permeability was indistinguishable from animals in the EAN group, and animals in the VGX/EAN or atropine/EAN group also had no protection against reducing intestinal permeability compared with animals in the EAN group (1542.36 ng/mL ± 370.61 ng/mL and 1645.87 ng/mL ± 312.33 ng/mL versus 1671.26 ng/mL ± 315.13 ng/mL). These data indicate that EA at ST36 can only offer protection to the gut in the presence of intact neurenteric innervation and may exert its protective effects via the cholinergic nerve pathway.

### 3.4. EA at ST36 Lowered Plasma DAO

**Figure 4:**
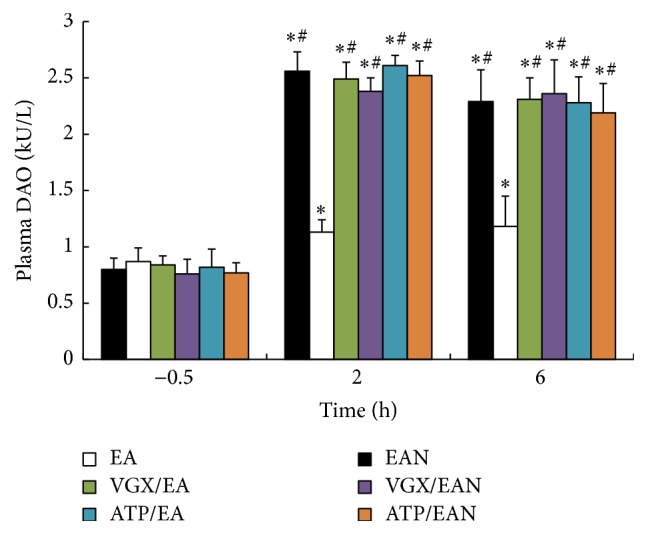
The level of plasma DAO was detected at −0.5, 2, and 6 h after scalds injury. Data are expressed as means ± SD (*n* = five animals at every time point per group). *∗* versus −0.5 h among the same group, *P* < 0.05; # versus EA group among 2 h and 6 h, *P* < 0.05.

Figure 4 illustrates the effect of EA at ST36 on the gut barrier function after 35% of TBSA scald injury by detecting the activity of diamine oxidase. Scald injury induced gut ischemia and had an impact on the gut barrier function. Animals in the EA group had a significantly lower level of plasma DAO when compared with the EAN group. EA at ST36 protected the gut barrier function after scalds injury, while vagotomy or intraperitoneal injection of atropine before EA at ST36 reversed its antiparalysis effects.

## 4. Discussion

The results of the present study indicate that EA at ST36 can successfully attenuate systemic inflammation, decrease gut permeability, and improve the mucosal blood flow in the small intestine, which is consistent with preserved intestinal barrier function after scald injury. The novel finding from this study is that EA at ST36 significantly attenuated the decrease of the intestinal impelling ratio after scalds injury which is important for physiological nutritional support in critical illnesses such as severe scald injuries [10]. These results provide further evidence for the role of acupuncture as a treatment of scald injury.

The gut plays a key role in the development of intestinal and systemic inflammatory response following severe scalds [11]. It becomes a source of proinflammatory mediators resulting from an impairment of the intestinal mucosal barrier that may amplify systemic inflammatory response syndrome (SIRS), develop a systemic response state and distant organ failure, and lead to multiple organ dysfunction syndrome (MODS) or even death [12–14]. The current treatment for scald shock focuses on maintaining a sufficient tissue perfusion and vital organ function with early and adequate fluid resuscitation [15]. However, intravenous fluid resuscitation is usually difficult to administer in austere environments. In recent years, we have been working on alternative methods to solve the complications caused by delayed fluid resuscitation and we have proved that EA at ST36 can significantly protect the intestinal barrier integrity and improve organ function and survival rate after fatal hemorrhagic shock in rats [2, 16].

Traditional Chinese medicine has been investigated with regard to whether it provides protective effects on intestinal and gastric mucosa after scald injury and hemorrhagic shock [17–19]. In this set of experiments, we stimulated the ST36 acupoints after scald injury, established its efficacy, and compared its effects with nonchannel acupoints after abdominal vagotomy or atropine injection. We demonstrated that EA at ST36 points is effective in alleviating gut dysfunction after 35% TBSA scald injury. Furthermore, we have shown that its biological effect is dependent on an intact vagus nerve.

Acupuncture has been mainly used in the treatment of inflammatory diseases, including asthma, rhinitis, inflammatory bowel disease, rheumatoid arthritis, epicondylitis, complex regional pain syndrome type 1, and vasculitis [20]. In a previous study, we have demonstrated that EA at ST36 can effectively protect organ function [21], improve the early survival rate, increase the intestinal tissue DAO activity, and alleviate intestinal ischemia in rats with delayed fluid replacement after hemorrhagic shock.

But there are few reports on the anti-inflammatory or therapeutic effects of acupuncture in scald wounds. In this study, EA at ST36 points effectively improved the mucosal blood flow in the small intestine of severely scalded rats, reduced the permeability of the distal ileum to 4-kDa FITC-dextran, and lowered Plasma DAO. We also disrupted the neurenteric axis via surgical abdominal vagotomy to prove that the protective effect of EA at ST36 depends on an intact vagus nerve.

Severe scalds also induce gut paralysis and delay gut emptying which would aggravate bacterial translocation and the local inflammatory response. Early enteral nutrition remains the mainstay of physiological nutritional support in critically ill patients after severe hemorrhage or burn injuries. Delayed gastrointestinal emptying affects or delays drug absorption. However, none of the commonly used drugs can reliably treat, that is, prevent, delayed gastrointestinal emptying currently [10]. Previous studies have found that acupuncture modulates important components involved in the pathogenesis of delayed gastrointestinal emptying [19, 22, 23]. It was also reported that acute postoperative nausea and vomiting (PONV) is related to the autonomic nervous system activity and may hence be amenable to acupuncture therapy [10]. Furthermore, several experimental studies in animals have demonstrated a possible modulating effect of acupoint stimulation on variables of gastrointestinal motility [24–27]. This study evaluates the effects of EA at ST36 on the intestinal impelling ratio after 35% of TBSA scald injury. It was found that scald injury induced gut paralysis and decreased the intestinal impelling ratio. EA at ST36 increased the intestinal impelling ratio 2 h and 6 h after scalds injury, while vagotomy or intraperitoneal injection of atropine before EA at ST36 reversed its antiparalysis effects. This is in accordance with previous studies and underlines the hypothesis that the mechanism of acupuncture treatment relies on an intact vagus nerve.

In summary, data from this study demonstrate that EA at ST36 acupoints had protective effects against burns-induced high intestinal permeability, plasma DAO, and low mucosal blood flow in the small intestine, which all contribute to barrier dysfunction in rats with severe scald injuries. EA at ST36 also increased the intestinal impelling ratio after scalds injury. The protective role of EA at the ST36 acupoints seems to be based on an intact vagus nerve and might exert its effects via the cholinergic *α*7 nicotinic acetylcholine receptor.

The present study also showed that acupuncture treatment offers an obvious active effect on the gut barrier and motility in severely scalded rats; this suggests that the therapeutic effect is directly related to the autonomic nerves. However, more studies are needed to confirm these results. Some details of the relationship between acupuncture treatment and an intact vagus nerve still need to be discovered. Furthermore, acupoint stimulation should also be attempted in patients with severe scald injury. Also, future studies should compare acupuncture and drug treatment, because the ultimate goal should be to delineate the best treatment for delayed gastric emptying in severe scald patients.

Acupuncture therapy has been used successfully in the fields of pain, gynecology, and obstetrics, among others, over the past decade, and its applications are growing. Severe injuries (such as scald and shock) are a promising but largely unexplored field for acupuncture therapy [28]. Acupuncture in cases of severe injury offers the possibility of an alternative nondrug treatment showing only few, nonserious side effects [29].

## 5. Conclusion

EA at Zusanli apparently protects the gut barrier and improves the intestinal motility as well as the mucosa blood flow in scalded rats; its mechanism is possibly related to the activation of the cholinergic nerve pathway.
